# ATAD3A: A Key Regulator of Mitochondria-Associated Diseases

**DOI:** 10.3390/ijms241512511

**Published:** 2023-08-07

**Authors:** Liting Chen, Yuchang Li, Alexander Zambidis, Vassilios Papadopoulos

**Affiliations:** Department of Pharmacology and Pharmaceutical Sciences, Alfred E. Mann School of Pharmacy and Pharmaceutical Sciences, University of Southern California, Los Angeles, CA 99089, USA; litingc@usc.edu (L.C.); yuchangl@usc.edu (Y.L.); zambidis@usc.edu (A.Z.)

**Keywords:** ATAD3A, mitochondria, mtDNA, mutation, mitochondrial respiration, cholesterol, neurological diseases, cancer

## Abstract

Mitochondrial membrane protein ATAD3A is a member of the AAA-domain-containing ATPases superfamily. It is important for the maintenance of mitochondrial DNA, structure, and function. In recent years, an increasing number of *ATAD3A* mutations have been identified in patients with neurological symptoms. Many of these mutations disrupt mitochondrial structure, function, and dynamics and are lethal to patients at a young age. Here, we summarize the current understanding of the relationship between ATAD3A and mitochondria, including the interaction of ATAD3A with mitochondrial DNA and mitochondrial/ER proteins, the regulation of ATAD3A in cholesterol mitochondrial trafficking, and the effect of known *ATAD3A* mutations on mitochondrial function. In the current review, we revealed that the oligomerization and interaction of ATAD3A with other mitochondrial/ER proteins are vital for its various functions. Despite affecting different domains of the protein, nearly all documented mutations observed in ATAD3A exhibit either loss-of-function or dominant-negative effects, potentially leading to disruption in the dimerization of ATAD3A; autophagy; mitophagy; alteration in mitochondrial number, size, and cristae morphology; and diminished activity of mitochondrial respiratory chain complexes I, IV, and V. These findings imply that ATAD3A plays a critical role in mitochondrial dynamics, which can be readily perturbed by *ATAD3A* mutation variants.

## 1. Introduction

ATPase family AAA-domain-containing protein 3 (ATAD3) is a member of the AAA+ superfamily. The AAA+ ATPases are a large family of proteins involved in various cellular processes, such as protein unfolding, protein degradation, DNA replication, and membrane fusion [1]. These proteins have a conserved ATPase domain that is responsible for ATP hydrolysis and the energy required for their functions [2]. ATAD3 has three paralogs, ATAD3A, ATAD3B, and ATAD3C. ATAD3B acts as a negative regulator of ATAD3A. In humans and other primates, all three paralogues are expressed, while mice and rats express only ATAD3A. ATAD3A is enriched in mitochondrial-associated membranes (MAMs) and was initially reported as a mitochondrial-DNA (mtDNA)-bound protein [3,4]. It binds to the displacement loop (D-loop) of mtDNA to regulate the distribution of mtDNA across mitochondrial membranes [3].

ATAD3A interacts with a complex network of mitochondrial proteins to regulate mitochondrial functions including respiration and quality control. Additionally, ATAD3A is crucial for the interaction between mitochondria and the endoplasmic reticulum (ER) in the regulation of ER stress, cholesterol transport, and cancer metastasis [5,6]. Moreover, ATAD3A regulates the mammalian target of rapamycin (mTOR), sterol regulatory element binding protein 1c (SREBP-1c), and cyclin D1 signaling pathways for cell proliferation [7], and its phosphorylation is regulated by insulin and serum, although the serum component mediating this effect has not been identified [8].

Numerous *ATAD3A* mutations have been identified in patients with neurological diseases. These mutations are distributed throughout different domains of the protein, resulting in dysfunctional ATAD3A. Patients exhibit disrupted mitochondrial structure and functions. Furthermore, ATAD3A has been associated with the sensitivity of the human body to anti-cancer drugs. This review aims to consolidate the existing knowledge regarding the regulatory mechanisms governing ATAD3A function in mitochondria and the impact of *ATAD3A* mutations on mitochondrial functionality in disease.

## 2. ATAD3A Structure

**Figure 1:**
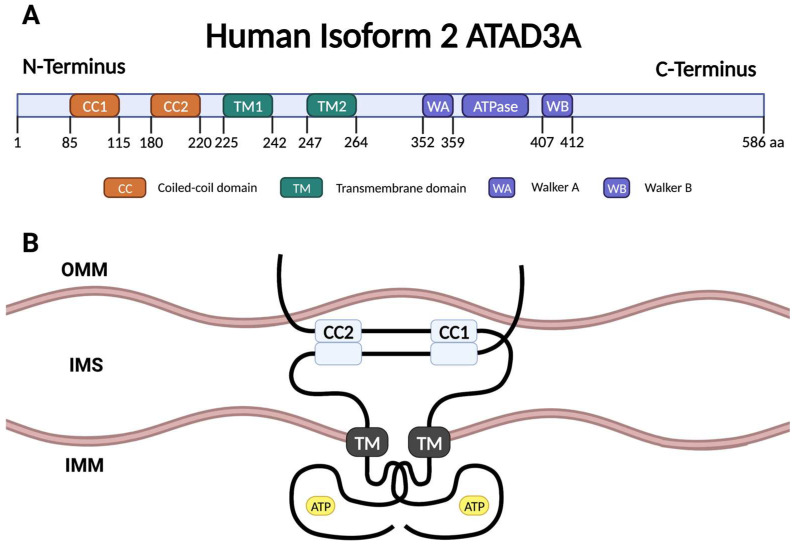
ATAD3A’s molecular structure and topology. (**A**) The 66 KD human isoform 2 ATAD3A protein labeled with the domains shown. The protein is composed of two coiled-coil domains, CC1 and CC2; two transmembrane domains, TM1 and TM2; a Walker A motif; an ATPase catalytic domain; and a Walker B motif. (**B**) Dimerized ATAD3A topology within the mitochondrial membranes. The two intermembrane regions of ATAD3A, CC1, and CC2, serve as a scaffold for oligomerization. The TM segments anchor ATAD3A in the IMM and direct the ATPase catalytic core towards the matrix. The N-terminus extends to the OMM. CC, coiled-coil; TM, transmembrane; WA, Walker A; WB, Walker B; IMM, inner mitochondrial membrane; OMM, outer mitochondrial membrane; IMS, intermembrane space.

Human *ATAD3A* is located on chromosome 1 at the 1p36.33 locus and has three transcript variants encoding three isoforms, with isoform 2 being the predominant one with 586 amino acids [9]. As depicted in Figure 1A, ATAD3A isoform 2 encompasses a structural composition that includes two coiled-coil domains, two transmembrane domains, a Walker A motif, a Walker B motif, and an ATPase domain [9]. The coiled-coil domains located in the N-terminal region are scaffolds for oligomerization [10]. The transmembrane segments anchor ATAD3A to the inner mitochondrial membrane (IMM) and position the ATPase catalytic core toward the matrix [10]. The C-terminal Walker A, Walker B, and ATPase domains collectively constitute the catalytic core, responsible for the hydrolysis of adenosine triphosphate (ATP) (Figure 1B) [11]. Compared to isoform 2, ATAD3A isoform 1 has an additional 48 amino acids in the coiled-coil domain 1, and isoform 3 lacks 79 amino acids in the N-terminus before the coiled-coil domain 1 [9].

## 3. ATAD3A and Mitochondrial Structure

### 3.1. ATAD3A and mtDNA

ATAD3A was originally characterized as a protein in mitochondrial nucleoids, which are complexes of mtDNA and proteins [3]. mtDNA encodes 13 proteins of the mitochondrial respiratory chain, so the replication and translation of mtDNA is important for mitochondrial oxidative phosphorylation [12]. mtDNA contains a D-loop, a short triple-stranded region that serves as a non-coding regulatory site for mtDNA replication and transcription because it contains promoters for mtDNA transcription and the origin of mtDNA replication [13,14]. The D-loop associates with the IMM and is the binding site for some nucleoid proteins. The binding of the mtDNA D-loop with nucleoid proteins is essential for the maintenance of mtDNA, nucleoid structure, and mtDNA transcription [12]. Functionally, it is still controversial whether nucleoids can move or traffic through the mitochondrial network [12,15,16]. Iborra et al. suggested that the movement of nucleoids in the mitochondrial matrix was limited because they were attached to the IMM and the movement was hindered by mitochondria cristae [15]. Ishihara et al. found that nucleoids could actively move to form clustered nucleoids, which was induced by defects in mitochondrial fission [16]. The study also suggested that dispersed nucleoids had high mtDNA transcription, whereas clustered nucleoids showed low mtDNA transcription, suggesting that nucleoid distribution is important for mtDNA transcription and, therefore, mitochondrial respiration [16].

**Figure 2:**
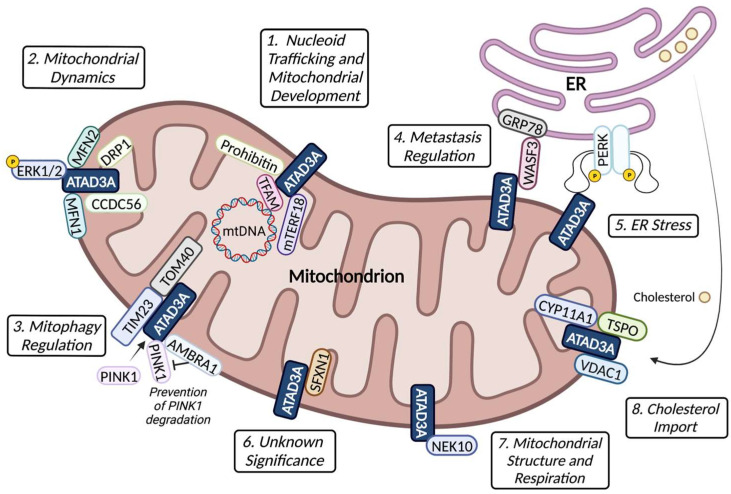
Schematic of ATAD3A interactions with mitochondrial proteins and mitochondrial activities. These interactions and activities include: (1) nucleoid trafficking by ATAD3A and prohibitin in response to the binding of the transcription factors mTERF18 and TFAM; (2) mitochondrial dynamics’ modulation through the binding of ATAD3A to ERK1/2, fission proteins DRP1 and CCDC56, and fusion proteins MFN1 and MFN2; the binding between ATAD3A and ERK1/2 is essential for the phosphorylation of ERK1/2; (3) mitophagy regulation by the coordination of ATAD3A, TIM23, and TOM40 for PINK1 degradation and AMBRA1 binding to ATAD3A for the prevention of PINK1 degradation; (4) cancer metastasis regulation mediated by the ATAD3A/GRP78/WASF3 complex; (5) ATAD3A binds to PERK to attenuate PERK signaling during ER stress; (6) ATAD3A also binds SFXN1, but its significance is unknown; (7) the regulation of mitochondrial structure and respiration by the binding of ATAD3A to NEK10; and (8) the import of cholesterol through the hormone-dependent mitochondrial metabolon formed by ATAD3A, TSPO, VDAC1, and CYP11A1.

As a nucleoid protein, ATAD3A assists mtDNA in forming or segregating mitochondrial nucleoids through the interaction of ATAD3A with the D-loop of mtDNA [3]. Silencing of ATAD3A results in the segregation or dissociation of D-loop-containing mtDNA held together by proteins, high mtDNA transcription, and enhanced formation of the mtDNA-coded proteins of the mitochondrial electron respiratory chain [3,16]. ATAD3A closely interacts with other mitochondrial nucleoid proteins, including mitochondrial transcription factor A (TFAM), prohibitin, and mitochondrial transcription termination factor 18 (mTERF18), to regulate the organization, distribution, and activities of nucleoids in the mitochondrial membrane (Figure 2 #1) [16,17,18]. TFAM is a mtDNA-binding protein important for the copy number of mtDNA. The decoration of TFAM on mtDNA renders the transcription of mtDNA less active [19]. The ATPase domain of ATAD3A binds to TFAM and regulates the trafficking of mitochondrial nucleoids, affecting mitochondrial size and number, the distribution of mitochondrial nucleoids, and respiratory complex formation [16]. Prohibitin is an IMM protein that maintains the copy number of mtDNA by stabilizing TFAM [20]. He et al. proposed that ATAD3A and prohibitin support nucleoids and mitochondrial translation at the IMM (Figure 2 #1) [17]. mTERF18 is a mitochondrial matrix protein that interacts with ATAD3A [18]. Kim et al. proposed that ATAD3A may directly interact with mTERF18 [18]. The study referenced the research of Frazier et al. showing that mutations of ATAD3A with compromised function resulted in disrupted nucleoid organization, reduced accumulation of complexes I, III, IV, and V, and decreased activity of complexes I and IV, which are effects shared by mTERF18 mutants, suggesting that they are both necessary for nucleoid organization and mitochondrial respiration (Figure 2 #1) [18,21]. The fine-tuned interactions of ATAD3A with other nucleoid proteins are vital for the normal distribution and expression of mtDNA, which further supports mitochondrial structure and biogenesis.

### 3.2. ATAD3A and Mitochondrial Cristae Morphology

ATAD3A plays a vital role in regulating the normal morphology of mitochondrial cristae, the internal membrane folds enriched in enzymes involved in the mitochondrial respiratory chain and ATP synthase [22]. Cristae membranes are connected to the mitochondrial inner boundary membrane through cristae junctions, which are essential for mitochondrial organization and function. In vivo, neuron- or skeletal-muscle-specific knockout of *Atad3a* in mice disrupted mitochondrial cristae morphology [6,22]. Similarly, in vitro, knockout of *ATAD3A* in HeLa cells or liver Huh7 cells also disrupted mitochondrial cristae morphology [23,24]. These findings support the significance of ATAD3A in regulating mitochondrial cristae organization and highlight its importance in maintaining mitochondrial function and structure in different cellular contexts. Remarkably, the skeletal-muscle-specific conditional knockout of *Atad3a* in mice exhibited a notable reduction in the quantity of mitochondrial cristae junctions. Conversely, in HeLa cells, the knockout of *ATAD3A* resulted in an increased presence of cristae junctions, coinciding with a global decrease in the overall abundance of cristae [6,23]. These contrasting observations raise intriguing questions regarding potential divergent functions of ATAD3A in distinct organs or species. Nonetheless, it is evident that ATAD3A plays an indispensable role in facilitating the formation of structurally intact mitochondrial cristae.

## 4. ATAD3A–Protein Interactions and Function

ATAD3A has been shown to form complexes with many proteins located in either the mitochondrial membrane or ER, indicative of its versatile role in mitochondria and at contact sites between mitochondria and the ER. Mitochondrial fission and fusion are two important processes of mitochondrial quality control, and ATAD3A has been reported to interact with multiple proteins involved in them in both humans and mice, including mitochondrial fission protein dynamin-related protein 1 (DRP1), fusion proteins mitofusin-1 (MFN1) and MFN2, and coiled-coil-domain-containing protein 56 (CCDC56), a positive regulator of mitochondrial fission (Figure 2 #2) [25,26,27]. Furthermore, the activation of extracellular signal-regulated kinase 1/2 (ERK1/2) mediated the activation of DRP1, and ATAD3A bound to ERK1/2 in the mitochondria of head and neck squamous cell carcinomas (HNSCC) cells activated the mitochondrial ERK1/2 signaling pathway (Figure 2 #2) [28,29]. These results suggest that ATAD3A may regulate DRP1 through ERK1/2 phosphorylation.

Jin et al. showed that ATAD3A was involved in the import of PINK1, a mitophagy initiator, to mitochondria for degradation in mouse hematopoietic progenitor cells [30]. ATAD3A coordinates with TOM40 at the OMM and TIM23 at the IMM to pass PTEN-induced kinase 1 (PINK1) through the mitochondrial membrane (Figure 2 #3). They showed that the deletion of ATAD3A in mouse hematopoietic cells induced increased mitophagy [30]. Furthermore, a pro-autophagic protein, the activating molecule in beclin-1-regulated autophagy (AMBRA1), can form a complex with ATAD3A and PINK1 at the OMM to regulate mitophagy [31]. Di Rienzo et al. showed that binding of AMBRA1 to ATAD3A prevented the degradation of PINK1 (Figure 2 #3) [31]. Interestingly, our recent study showed that the knockout of *ATAD3A* using Crispr/Cas9 genetic editing in the human liver cancer cell line HuH7 dramatically reduced levels of PINK1 [24], which suggests that ATAD3A interacts with PINK1 differently in different cells.

In addition, ATAD3A also interacts with proteins located in the ER. ATAD3A can form a complex with GRP78 and WASF3 (Figure 2 #4) [5]. GRP78 is an ER chaperone protein regulating ER protein folding. WASF3 is a protein involved in cancer cell metastasis. ATAD3A and GRP78 together stabilize WASF3 at the mitochondrial membrane, promoting cancer metastasis [5,32]. Moreover, the N-terminus of ATAD3A directly interacts with the kinase insert loop of protein-kinase-R-like endoplasmic reticulum kinase (PERK), an ER stress senor kinase located in ER, attenuating the PERK-mediated signaling during ER stress (Figure 2 #5) [33].

Sideroflexin 1 (SFXN1), a human mitochondrial serine transporter, was also shown to interact with ATAD3A in MCF7 cells, a human breast cancer cell line, identified by co-immunoprecipitation followed by shotgun mass spectrometry (coIP-MS) (Figure 2 #6) [34]. The significance of this interaction is yet to be understood. Furthermore, ATAD3A in nerves was shown to be a potential interactor with mitosis-gene-A-related kinase 10 (NEK10), which is involved in the regulation of the mitochondrial structure, citrate synthase activity, respiratory chain complexes, and cell death (Figure 2 #7) [35]. Additional functions for ATAD3A in mitochondria are likely.

## 5. ATAD3A and Cholesterol Homeostasis

The role of ATAD3A in the regulation of cholesterol metabolism has been reported in human or mouse steroidogenic cell lines, disease models, and human liver cancer cell lines. In NCI-H295R cells, a human steroidogenic cell line, ATAD3A was upregulated upon stimulation with steroidogenic hormone and helped the formation of contacts sites between the OMM and IMM to facilitate the import of cholesterol [10]. Similarly, in the steroidogenic MA-10 mouse Leydig cell line, ATAD3A was shown to regulate the cholesterol transport at the mitochondrial membrane, which was dependent on the proper insertion of ATAD3A into the mitochondria and ER by the 50 amino acids in the N-terminus [4,36]. The deletion of ATAD3A or its N-terminus reduced steroid production and progesterone biosynthesis, respectively, upon hormonal stimulation, suggesting a role for ATAD3A in the transport of cholesterol for metabolism into the mitochondria [4]. Furthermore, in steroidogenic Leydig cells, ATAD3A forms a hormone-dependent mitochondrial metabolon with the OMM translocator protein (TSPO), voltage-dependent anion channel (VDAC), and IMM protein cytochrome P450 family 11 subfamily A member 1 (CYP11A1) to transport cholesterol used for steroid formation through the mitochondrial membranes to CYP11A1 (Figure 2 #8) [36]. In addition, we recently showed that, when Huh7 cells, a liver cancer cell line, were overloaded with excessive free cholesterol, total cholesterol levels were substantially higher when *ATAD3A* was knocked out [24]. Along with the steroidogenic cell results, these data show that ATAD3A is necessary for the transport and metabolism of cholesterol.

ATAD3A may also serve a different role in cholesterol transport and metabolism under certain disease conditions. Zhao et al. recently showed that, in mouse models of Alzheimer’s disease, ATAD3A oligomers were increased and induced cholesterol accumulation at the MAM by inhibiting CYP46A1, the rate-limiting enzyme for brain cholesterol metabolism [37]. Suppression of ATAD3A oligomerization restored levels of CYP46A1 [37]. In that study, ATAD3A negatively regulated the transport and metabolism of cholesterol. Taken together, the opposite roles of ATAD3A in the transport and metabolism of cholesterol could be attributed to different roles for ATAD3A in different cells or different roles for ATAD3A in response to environmental changes.

ATAD3A also regulates cholesterol transport from the plasma membrane to the ER. Cholesterol is released from low-density lipoprotein (LDL) and then transported to the plasma membrane. When there is excessive cholesterol in the plasma membrane, cholesterol is transferred to the ER, where cholesterol is esterified and stored in lipid droplets (LDs) [38]. *ATAD3A* skeletal-muscle-specific conditional KO in mice decreased the cholesterol ester/free cholesterol ratio, indicative of a reduction in cholesterol transference to the ER [6].

## 6. ATAD3A Mutations, Mitochondrial Abnormalities and Neurological Diseases

### 6.1. ATAD3A Point Mutations

**Figure 3:**
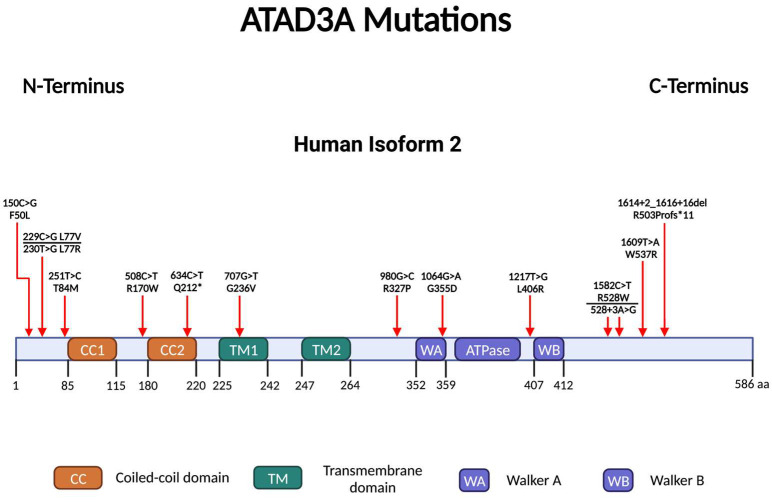
Point mutations of ATAD3A reported to date. Point mutations are labeled using the molecular structure of ATAD3A.

In the past decade, many *ATAD3A* mutations were reported to be associated with different disease phenotypes and with different impacts on the ATAD3A protein and mitochondria. To date, reported mutations of *ATAD3A* are located in different domains of the protein, such as the Walker A/B motif, N-terminus, and C-terminus domains (Figure 3). The influence of these mutations on the protein and mitochondria is described individually in the following section and summarized in Table 1. Generally, the majority of the point mutations are either loss-of-function or dominant-negative and lead to reduced expression of ATAD3A [21,39,40,41,42,43,44]. These point mutations may also induce autophagy and mitophagy, disrupt mitochondrial cristae morphology or content, and reduce the expression or activity of complexes I, IV, and V [39,40,41,45,46,47]. Nonetheless, the exact presentation of mitochondrial content and size is divergent between different point mutations, suggesting the complexity of ATAD3A in the regulation of mitochondrial dynamics [39,40,41,45,46,48]. Moreover, the effect of the same mutation on the mitochondria differs in different cell types [47]. The copy number variants in *ATAD3A* usually result in the generation of fusion proteins ATAD3A-ATAD3B or ATAD3A-ATAD3C, which may induce the aggregation of mitochondria and/or mtDNA, decrease mtDNA synthesis, increase free cholesterol, interfere with the oligomerization of the ATAD3A protein, and reduce the activity of complexes I and IV [21,49,50].

#### 6.1.1. Walker A/B Motif

In 2017, Cooper et al. reported a dominantly inherited heterozygous variant, c.1064G>A (p.Gly355Asp), in *ATAD3A* [39]. It is a dominant-negative variant that affects the Walker A motif and reduces ATPase activity. In neurons derived from the reprogramming and differentiation of patient’s fibroblasts, mitochondria were fragmented and accumulated in the cells, but the mtDNA copy number was not affected. Additionally, basal autophagy was increased. Overexpression of the ATAD3A G355D mutant in primary human fibroblasts also induced mitochondrial fragmentation and accumulation of lysosomes.

In 2019, Peralta et al. identified a novel recessive missense variant in *ATAD3A*, c.1217T>G (p.Leu406Arg), from four siblings with fatal neonatal cerebellar hypoplasia from a consanguineous family [45]. This variant resulted in reduced ATAD3A expression. In the fibroblasts of these patients, mitochondrial cristae content and mitochondrial size were reduced, confirming the important role of ATAD3A in mitochondrial biogenesis.

In 2021, c.980G>C (p.Arg327Pro), a *ATAD3A* mutation adjacent to the Walker A motif, was identified by Yap et al. in three siblings with congenital cataract, hypertrophic cardiomyopathy, and elevated 3-methylglutaconate in urine [40]. A *Drosophila* mutation R333P, homologous to the human c.980G>C (p.Arg327Pro), was created for the functional studies of the human allele. R333P is a severe loss-of-function allele, which moderately reduced ATAD3A protein expression in the head of *Drosophila* and increased mitochondrial content and size.

#### 6.1.2. C-Terminus

Harel et al. found a recurrent de novo *ATAD3A* c.1582C>T (p.Arg528Trp) variant in five unrelated individuals who displayed neurological phenotypes, including global developmental delay, hypotonia, optic atrophy, axonal neuropathy, and hypertrophic cardiomyopathy [41]. The disease showing these phenotypes was named Harel-Yoon syndrome in other publications thereafter. The c.1582C>T (p.Arg528Trp) variant is a dominant-negative variant that triggers mitophagy and reduces mitochondrial size and content. Recently, Lepelley et al. further examined this variant and the c.1064G>A (p.Gly355Asp) identified by Cooper et al., two dominant-negative mutants of *ATAD3A* [53]. Six out of seven patients with one of these two mutations had enhanced interferon-stimulated gene (ISG) expression in the blood, suggesting a potential link between ATAD3A and interferon signaling. The study showed that loss of ATAD3A led to the release of mtDNA into the cytoplasm, which activated cyclic GMP-AMP synthase (cGAS) and the stimulator of interferon genes (STING) and enhanced interferon signaling.

Another C-terminal mutant, a homozygous *ATAD3A* 528+3A>G variant, was discovered by Hanes et al. in a girl with Harel-Yoon syndrome from healthy and consanguineous parents [42]. 528+3A>G is a loss-of-function variant resulting in the retention of intron 3 and premature termination at the 3′ end of exon 3. Neurodevelopment of the patient apparently started to regress at 5–8 months of age. The expression of ATAD3A was decreased in the fibroblasts of the patient, but no other biochemical analysis was provided.

Dorison et al. identified two novel *ATAD3A* mutations simultaneously present in two siblings with axonal sensory–motor neuropathy and neonatal cataract from healthy non-consanguineous parents [46]. One mutation was a c.1609T>A (p.Trp537Arg) point mutation inherited from the mother, and the other was an intronic 15 bp deletion c.1614+2_1616+16del (p.Arg503Profs*11), resulting in premature termination inherited from the father. Mutations resulted in reduced ATAD3A expression in the fibroblasts of the patients, but did not affect mitochondrial morphology in these cells. In contrast, an abnormal mitochondrial morphology, increased number and size of mitochondria, disrupted cristae network, and decreased activity of complex I were observed in the skeletal muscles of both patients. These findings suggest that the impact of the same *ATAD3A* mutation is highly divergent on different cell types and reveal the diversity of the regulation of ATAD3A on mitochondria in different cell types.

#### 6.1.3. N-Terminus

In 2017, Peeters-Scholte et al. discovered two *ATAD3A* mutations, c.230T>G (p.Leu77Arg) and c.634C>T (p.Gln212*), which simultaneously existed in two male siblings with abnormalities in the basal ganglia and white matter and corneal clouding, born from non-consanguineous parents [48]. c.230T>G (p.Leu77Arg) is a missense mutation located in the N-terminal sequence before coiled-coil 1. c.634C>T (p.Gln212*) results in the premature termination of the ATAD3A protein at the coiled-coil 2 domain. One of the two siblings carrying these two mutations was reported to have increased amounts of mitochondria in the cardiomyocytes. Considering the two mutations existing concomitantly, this obscured which mutation was the dominant cause of the mitochondrial abnormality in the cardiomyocytes.

Al Madhoun et al. first identified an *ATAD3A* c.251T>C (p.Thr84Me) biallelic variant in a female with developmental delay and cerebellar atrophy (similar to Harel-Yoon syndrome without cardiomyopathy or optic atrophy) from consanguineous parents in 2019 [43]. The mutation was adjacent to the N-terminal coiled-coil 1 domain of ATAD3A, which is important for the interaction of mitochondria with other organelles. They speculated that the variant may generate a non-functional protein, but biochemical analysis was not provided in the publication. Recently, Chen et al. reported a heterozygous c.251T>C (p.Thr84Me) and exon 1–2 deletion in the *ATAD3A* gene in a boy from non-consanguineous parents [54]. The patient also showed Harel-Yoon syndrome, including refractory epilepsy, hypotonia, global developmental delay, and congenital cataract [54]. Biochemical studies on the protein or mitochondria were not presented.

In 2021, Yap et al. reported four *ATAD3A* missense mutations, c.229C>G (p.Leu77Val), c.150C>G (p.Phe50Leu), c.508C>T (p.Arg170Trp), and c.707G>T (p.Gly236Val), in eight patients with developmental delay or neurological symptoms from five families [40]. c.229C>G (p.Leu77Val) and c.150C>G (p.Phe50Leu) are adjacent to the coiled-coil 1 domain. c.508C>T (p.Arg170Trp) is close to the coiled-coil 2 domain. c.707G>T (p.Gly236Val) is in the transmembrane domain. The functional studies of these mutations were performed in *Drosophila* carrying mutations homologous to the human mutations identified. Based on the results obtained from *Drosophila*, c.150C>G (p.Phe50Leu) and c.707G>T (p.Gly236Val) seemed to be loss-of-function alleles. Of note, c.707G>T (p.Gly236Val), but not the other three mutations, resulted in complete loss of the ATAD3A protein. c.229C>G (p.Leu77Val) and c.508C>T (p.Arg170Trp) were partial loss-of-function alleles that may cause smaller mitochondria and loosened and fragmented cristae. In 2023, Skopkova et al. identified four more patients from two families carrying heterozygous c.229C>G (p.Leu77Val) and exon 3–4 deletion in the *ATAD3A* gene [47]. The clinical picture of these patients was similar to the patient carrying the c.229C>G (p.Leu77Val) variant reported by Yap et al. in 2021. The expression of ATAD3A was profoundly reduced in the fibroblasts of the patients, without observable changes in the expression of ATAD3B. In one patient out of the four, a reduction in the activity of complex IV, the levels of subunits COX2 and complex V, and the mitochondrial proteosynthesis rate were discovered in the fibroblasts. Furthermore, the muscle mitochondria of the patient showed reduced protein expression of complexes I, IV, and V.

### 6.2. Copy Number Variants

Desai et al. reported five individuals who had biallelic deletions in the region encoding the *ATAD3C*, *ATAD3B*, and *ATAD3A* genes [49]. These deletions resulted in the generation of an ATAD3A-ATAD3B chimeric protein. ATAD3A expression was downregulated in the fibroblasts of these patients, and the fibroblasts from some of them showed enlarged mtDNA foci, suggestive of aggregated mtDNA; slow mtDNA synthesis; and an increase of free cholesterol.

Two de novo intergenic duplications of the *ATAD3A* gene cluster were reported in five unrelated neonates by Gunning et al. [50]. The duplication resulted in the generation of a fusion gene of *ATAD3A* and *ATAD3C* that was stably expressed, as well as a duplication of *ATAD3B*. In the fibroblasts of these patients, the aggregation of mitochondria, the accumulation of mtDNA, and an increase in free cholesterol were observed. These observations by Gunning et al. were similar to those associated with *ATAD3* cluster deletions reported by Desai et al. [34], indicating that the protein resulting from the fusion of the ATAD3A and *ATAD3C* genes may be nonfunctional.

Azova et al. identified a heterozygous de novo duplication of the *ATAD3* gene cluster in a male infant with multiple congenital anomalies, left ventricular hypertrophy, and neurological dysfunction [51]. The infant developed endogenous Graves’ disease at five months of age. Graves’ disease is an autoimmune disease that can lead to hyperthyroidism characterized by excessive production of thyroid hormone and is uncommon in children under five years old [51,55]. Azova et al. suspected a possible mitochondrial dysfunction that may potentially account for a novel pathophysiology for early-onset Graves’ disease [51,56,57].

Frazier et al. described seventeen patients from sixteen families who all carried a recurrent de novo 68 kbp duplication in the *ATAD3* locus [21]. The duplication was located at different breakpoints of *ATAD3A* and *ATAD3C* in different patients, but all resulted in the duplication of *ATAD3B* and the generation of chimeric ATAD3A-ATAD3C proteins. The chimeric ATAD3A-ATAD3C proteins had dominant-negative effects and disturbed the oligomerization of ATAD3A. The expression of complexes I, III, IV, and V was decreased in the heart, and the activity of complexes I and IV was decreased in the liver and heart of these patients.

In 2022, Ebihara et al. reported two female siblings with spinal cord hypoplasia who had inherited a heterozygous *ATAD3A* deletion, a 38 kbp *ATAD3B/3A* deletion in the paternal allele and a 19 bp deletion in *ATAD3A* exon 6 in the maternal allele [52]. The 19 bp deletion was expected to be a null allele since it induced premature termination of the ATAD3A protein. The mitochondrial respiratory chain enzyme activity in the liver was normal in both patients. One patient had complex I deficiency in the myocardium, but mitochondrial respiratory chain enzyme activity in the myocardium from the other patient was not tested due to material limitations.

Tawfik et al. identified a homozygous mutation, c.624_644del (p.Glu209_Glu215del), in the *ATAD3A* gene in an 11-year-old male with Harel-Yoon syndrome, accompanied by novel features such as fatigable ptosis, facial weakness, progressive bulbar palsy, and obsessive–compulsive disorder [44]. The 20 bp deletion resulted in the generation of a loss-of-function *ATAD3A* variant. Mitochondrial dysfunction was suggested, supported by an elevated lactate peak on magnetic resonance spectroscopy, but biochemical assays were not provided.

## 7. ATAD3A and Mitochondria in Cancer

### 7.1. ATAD3A Expression and Cancer

ATAD3A was reported to be upregulated in certain types of cancer, including hepatocellular carcinoma (HCC), head and neck squamous cell carcinoma (HNSCC), and lung adenocarcinoma [29,58,59,60]. Interestingly, in HCC and lung adenocarcinoma, high expression of ATAD3A was correlated with severity of disease, poor prognosis, and low survival rates [58,59]. However, in breast cancer, low expression of ATAD3A was associated with shorter overall survival compared to high expression of ATAD3A [61]. This suggests that the role of ATAD3A varies in different types of cancer.

### 7.2. ATAD3A, Mitochondria and Drug Resistance

ATAD3A has been shown to positively regulate chemotherapy drug resistance in prostate cancer, lung adenocarcinoma, glioblastoma multiforme (GBM), and uterine cervical cancer [62,63,64,65]. Silencing of ATAD3A in cancer cell lines led to mitochondrial fragmentation and/or increased chemotherapy drug sensitivity [29,63].

Not only does ATAD3A promote the resistance of cancer to chemotherapy, but it also negatively affects the therapeutic response of cancer patients to the immune checkpoint inhibitor targeting PD-1/PD-L1 signaling used in combination with chemotherapy [66]. PD-1 is a ligand expressed in T cells, and PD-L1 is a receptor on the tumor cell surface [67]. The PD-1/PD-L1 system helps tumor cells block anti-tumor immune responses [67]. Immune checkpoint inhibitor therapy exerts anti-tumor effects by disrupting the binding of PD-1 and PD-L1 [68]. PD-L1 can be recruited to mitochondria from the cell surface and degraded by PINK1-mediated mitophagy [66]. High expression of ATAD3A inhibits PINK1-mediated mitophagy, blocking the degradation of PD-L1 and accumulating PD-L1 on the cell surface. This suggested that ATAD3A is a promising target to increase the therapeutic response to the combination of immunotherapy and chemotherapy [66].

## 8. Conclusions

ATAD3A function is regulated through oligomerization and interactions with numerous proteins in both the mitochondria and ER. These protein–protein interactions are complex and crucial for ATAD3A’s participation in multiple cellular processes. Mutations in ATAD3A may compromise mitochondrial structure, mtDNA distribution and number, the activity of respiratory chain complexes, and ATAD3A oligomerization. Different mutations in *ATAD3A* exhibit both common and/or specific mitochondrial abnormalities unique to each mutation. Point mutations usually serve as null alleles or exert dominant-negative effects. However, different point mutations have different tissue specificities, and not every tissue is affected to an equal extent even by the same mutation. Although it is predicted that *ATAD3A* point mutations disrupt normal mitochondrial structure and function, the exact impact of each mutation on mitochondrial number, size, and respiratory chain activity is highly divergent and difficult to predict.

Deciphering the precise functions, regulation, and interactions of different domains or even each mutation of ATAD3A at the mitochondrial membrane can be challenging. However, understanding the major impact of *ATAD3A* mutations on mitochondria can assist in the development or selection of potential therapies.

## 9. Perspective

ATAD3A plays a critical role in the maintenance of mitochondrial function and lipid homeostasis. Studies in recent decades have shed light on its complexity in the regulation of diverse mitochondrial events and lipid homeostasis. There is a strong link between the dysregulation of ATAD3A and diseases such as neurodegenerative disorders and cancer. The important role of ATAD3A in lipid homeostasis also indicates a relationship between ATAD3A and lipid-related diseases such as fatty liver diseases. Therefore, ATAD3A could be a potential therapeutic target for multiple human diseases. However, direct evidence from testing the effects of the modulation of ATAD3A on the progression of these diseases is lacking.

In the future, studies are needed to not only fully elucidate the mechanisms underlying ATAD3A’s functions and its role in disease pathogenesis, but also to seek confirmatory evidence to rescue damage caused by ATAD3A abnormalities. Investigation into the interactions between ATAD3A and other mitochondrial proteins could also reveal potential targets for drug development. Overall, ATAD3A’s pivotal role in the regulation of mitochondria-related activities and its implications in diverse diseases make it a promising area for future research on both mitochondrial and metabolic diseases.

Mutations in *ATAD3A* have been frequently reported, but functional studies on *ATAD3A* mutations are hindered due to limited accessibility to suitable human tissue materials. To overcome this, a few recent studies used *Drosophila* to perform functional studies of mutations homologous to human *ATAD3A* point mutations, which may help predict the function of human *ATAD3A* point mutations. Researchers may take advantage of this model to gather more knowledge about human *ATAD3A* point mutations discovered to date or newly identified in the future, especially with limited access to appropriate human samples.

## Figures and Tables

**Table 1:** The effects of ATAD3A mutations on the protein and mitochondria.

Type	Protein Domain	Name	Effects				Diseases/Symptoms	Ref.
			Nature	ATAD3A Protein	Mitochondria			
					Cell Type	Effects		
Point mutations	Walker A motif	c.1064G>A (p.Gly355Asp)	Dominant-negative	Reduced ATPase activity	Neuron differentiated from fibroblast	Fragmentation and accumulation of mitochondria, increase of autophagy	Hereditary spastic paraplegia (HSP) and axonal neuropathy and dyskinetic cerebral palsy	[39]
	Adjacent to Walker A motif	c.980G>C (p.Arg327Pro) *Drosophila p.Arg333Pro*	*Loss-of-function*	*Reduced expression in head*	*Embryo*	*Increase in mitochondrial content and size*	Congenital cataract, hypertrophic cardiomyopathy, and elevated 3-methylglutaconate in urine	[40]
	Adjacent to Walker B motif	c.1217T>G (p.Leu406Arg)	N/A *	Reduced expression in fibroblasts	Fibroblast	Reduction in mitochondrial cristae content and mitochondrial size	Fatal neonatal cerebellar hypoplasia, seizures, axial hypotonia, hypertrophic cardiomyopathy, hepatomegaly, congenital cataract, and dysmorphic facies	[45]
	C-terminus	c.1582C>T (p.Arg528Trp)	Dominant-negative	N/A	Fibroblast	Reduction of mitochondrial content (not significant) and elevation of mitophagy	Global developmental delay, hypotonia, optic atrophy, axonal neuropathy, and hypertrophic cardiomyopathy (Harel-Yoon syndrome)	[41]
		528+3A>G	Loss-of-function	Premature termination and reduced expression in fibroblasts	N/A	N/A	Harel-Yoon syndrome	[42]
		c.1609T>A (p.Trp537Arg) and c.1614+2_1616+16del (p.Arg503Profs*11) †	Not dominant-negative for p.Arg503Profs*11	Premature termination for p.Arg503Profs*11 and reduced expression in fibroblasts	Skeletal muscle	Abnormal mitochondrial morphology, increased number and size of mitochondria, disrupted cristae network, and decreased activity of complex I	Axonal sensory–motor neuropathy and neonatal cataract	[46]
	N-terminus	c.230T>G (p.Leu77Arg) and c.634C>T (p.Gln212*) †	N/A	Premature termination for c.634C>T (p.Gln212*)	Cardiomyocyte	Increased amount of mitochondria	Abnormalities in basal ganglia and white matter, corneal clouding, cataract, GFAP-positive gliosis in retina, and depletion of ganglion cells in retina	[48]
		c.251T>C (p.Thr84Me)	Potential loss-of-function	N/A	N/A	N/A	Harel-Yoon syndrome without cardiomyopathy or optic atrophy	[43]
		c.229C>G (p.Leu77Val) *Drosophila p.Leu83Val*	*Partial loss-of-function*	*Unaffected expression in head*	*Larvae*	*Unaffected mitochondrial content*	Global developmental delay, congenital cataract, and hypertrophic cardiomyopathy	[40]
					*Adult muscle*	*Smaller mitochondria, loosened and fragmented cristae, and potentially increased autophagy and mitophagy*		
		c.150C>G (p.Phe50Leu) *Drosophila p.Phe56Leu*	*Loss-of-function*	*Unaffected expression in head*	*Embryo*	*Abnormal increase in mitochondrial content and size*	Hypotonia, cloudy corneas, hypertrophic cardiomyopathy, cerebellar atrophy/hypoplasia, and elevated 3-methylglutaconate in urine	
		c.508C>T (p.Arg170Trp) *Drosophila p.Arg176Trp*	*Partial loss-of-function*	*Unaffected expression in head*	*Larvae*	*Unaffected mitochondrial content*	Moderate–severe learning difficulties, ataxia, muscle wasting, cerebellar atrophy/hypoplasia, and hearing loss	
					*Adult muscle*	*Smaller mitochondria, loosened and fragmented cristae, and potentially increased autophagy and mitophagy*		
		c.707G>T (p.Gly236Val) *Drosophila p.Gly242Val*	*Loss-of-function*	*Complete loss of protein in head*	*Embryo*	*Abnormal increase in mitochondrial content and size*	Central hypotonia, increased peripheral tone, hypertrophic cardiomyopathy, and cerebellar atrophy/hypoplasia	
		c.229C>G (p.Leu77Val) and exon 3–4 deletion †	N/A	Reduced expression in fibroblasts	Fibroblast	Reduction in the activity of complex IV, levels of subunits COX2 and complex V, and mitochondrial proteosynthesis rate	Bilateral congenital cataracts, strabismus, residual nystagmus, ophthalmoplegia, hypotonia, hyporeflexia, axonal sensory–motor neuropathy, growth delay, cerebellar syndrome, etc.	[47]
					Muscle	Reduction in protein expression of complexes I, IV, and V		
Copy number variants	N/A	Biallelic deletions in the region encoding the ATAD3C, ATAD3B and ATAD3A genes	N/A	ATAD3A-ATAD3B chimeric protein and reduced ATAD3A expression in fibroblasts	Fibroblast	Aggregation and enlargement of mtDNA, slow mtDNA synthesis, and increase of free cholesterol	Fatal congenital pontocerebellar hypoplasia	[49]
		Intergenic duplications of ATAD3A gene cluster	N/A	ATAD3A-ATAD3C chimeric protein (maybe nonfunctional) and ATAD3B duplication	Fibroblast	Aggregation of mitochondria, accumulation of mtDNA, and increase of free cholesterol	Lethal metabolic disorder characterized by cardiomyopathy, corneal opacities, encephalopathy, hypotonia, and seizures	[50]
		Duplication of the ATAD3 gene cluster	N/A	N/A	N/A	N/A	Graves’ disease	[51]
		68 kbp duplications in ATAD3 locus	Dominant-negative	ATAD3A-ATAD3C chimeric protein	Liver and heart	Disturbance of the oligomerization of ATAD3A and reduction of the activity of complexes I and IV Heart only: decreased expression of complexes I, III, IV, and V	Lethal perinatal cardiomyopathy, persistent hyperlactacidemia, frequent corneal clouding or cataracts, and encephalopathy	[21]
		38 kbp ATAD3B/3A deletion and 19 bp deletion in ATAD3A exon 6 †	Potential loss-of-function	Premature termination for 19 bp deletion in ATAD3A exon 6	Myocardium	Complex I deficiency	Severe spinal cord hypoplasia and pontocerebellar hypoplasia	[52]
					Liver	Normal respiratory chain enzyme activity		
		c.624_644del (p.Glu209_Glu215del)	Loss-of-function	N/A	N/A	Suggested mitochondrial dysfunction	Harel-Yoon syndrome, fatigable ptosis, facial weakness, progressive bulbar palsy, and obsessive-compulsive disorder	[44]

Note: Normal text is for observations associated with mutations in humans, while italicized text is for observations associated with homologous mutations in *Drosophila*. * N/A, not applicable. †, two mutations simultaneously exist.

## Data Availability

Not applicable.

## Abbreviations

ATAD3A	ATPase family AAA-domain-containing protein 3A
ATP	Adenosine triphosphate
AMBRA1	Beclin-1-regulated autophagy
CCDC56	Coiled-coil domain-containing protein 56
cGAS	Cyclic GMP-AMP synthase
coIP-MS	Co-immunoprecipitation followed by shotgun mass spectrometry
CYP11A1	Cytochrome P450 family 11 subfamily A member 1
D-loop	Displacement loop
DRP1	Dynamin-related protein 1
GBM	Glioblastoma multiforme
GRP78	Glucose-regulated protein
HCC	Hepatocellular carcinoma
HNSCC	Head and neck squamous cell carcinomas
IMM	Inner mitochondrial membrane
ISG	Interferon-stimulated gene
LDL	Low-density lipoprotein
LDs	Lipid droplets
MAMs	Mitochondrial-associated membranes
MFN1	Fusion proteins mitofusin-1
mtDNA	Mitochondrial DNA
mTERF	Mitochondrial transcription termination factor
mTOR	Mammalian target of rapamycin
NEK10	NIMA-related kinase 10
OMM	Outer mitochondrial membrane
PERK	Protein-kinase-R-like endoplasmic reticulum kinase
SFXN1	Sideroflexin 1
SREBP-1c	Sterol regulatory element binding protein 1c
STING	Stimulator of interferon genes
TFAM	Mitochondrial transcription factor A
TIM23	Translocase of inner mitochondrial membrane 23
TOM40	Outer mitochondrial membrane 40
TSPO	Translocator protein
VDAC	Voltage-dependent anion channel
WASF3	Wiskott-Aldrich syndrome protein family member 3
